# Synthesis of silver nanoparticles employing *Polyalthia longifolia* leaf extract and their *in vitro* antifungal activity against phytopathogen

**DOI:** 10.1016/j.bbrep.2022.101320

**Published:** 2022-08-12

**Authors:** Alankrita Dashora, Kavita Rathore, Shani Raj, Kanika Sharma

**Affiliations:** Department of Botany, Mohanlal Sukhadia University, Udaipur, Rajasthan, India

**Keywords:** Silver nanoparticle, *Polyalthia longifolia*, Green synthesis, Antifungal activity, Cyto-morphology

## Abstract

The *P. longifolia* mediated silver (PL-AgNPs) nanoparticles are very stable and efficient. UV–Vis spectroscopy, dynamic light scattering (DLS), X-ray diffraction (XRD), transmission electron microscope (TEM), scanning electron microscope (SEM), and energy dispersive X-ray spectroscopy (EDX) were used to characterize the produced AgNPs. UV–Vis analysis showed a characteristic peak at 435 nm corresponding to surface plasmon resonance. The synthesis process was spectrophotometrically optimized for various parameters. After optimization, highly stable AgNPs were prepared using 3.0 ml of *P. longifolia* leaf extract, pH 7.0, 1.0 mM AgNO_3_, and 60 °C. The zeta potential was measured by DLS, which showed −20.8 mV and the PDI value was 5.42. TEM and SEM analysis shows a spherical shape of the synthesized nanoparticles, and the size was measured between 10 and 40 nm. EDX analysis showed intense peaks from silver and oxygen and small peaks from various metal atoms such as Na, P, S and Al indicating their presence in trace amounts. The average size of the PL-AgNPs was 14 nm. The phytochemical analysis shows that the presence of alkaloids, essential oils and saponins seems to be responsible for the synthesis of nanoparticles. PL-AgNPs were further investigated for their antifungal activity against *Alternaria alternata*. The minimum inhibitory concentration (MIC), minimum fungicidal concentration (MFC) and effect of nanoparticles on cytomorphology of *A. alternata* have also been reported. Biosynthesized nanoparticles have proven to be inexpensive, environmentally friendly, stable, easily reproducible, and highly effective against plant-pathogenic fungi.

## Highlights

• Green synthesis of AgNPs using aqueous leaf extract of *Polyalthia longifolia.*
• Characterization using UV–vis, DLS, XRD, TEM, SEM, EDX.
• Optimization of AgNPs at different Temperature, pH, Concentration and Time.
• Nanoparticles were stable for more than 5 months.
• The antifungal activity of the AgNPs against *A. alternata* were studied.

## 1 Introduction

Nanotechnology has received tremendous attention over the past few decades due to its wide application in various fields. Nanoparticles are the basis in nanotechnology and have far-reaching applications compared to those of bulk counterparts [1]. Due to their large surface-to-volume ratio and their crystallographic surface structure, they have increased chemical activity [2]. It has been observed that certain carbon-based nanomaterials [[3], [4], [5]] such as graphene oxide nanoparticles, carbon nanotubes and fullerenes, metallic nanoparticles, magnetite nanoparticles, etc. have potent antimicrobial properties [6]. Metallic nanoparticles are mainly made of gold, silver, platinum and lead [7]. Among metals, silver is widely recognized for its role as a catalyst in various antimicrobial activities, surface-enhanced Raman scattering [8], healthcare, and sensors [9]. Its inhibitory action is shown against microorganisms present in various medical and industrial processes. They penetrate the cells of microbes by attaching themselves to the cell wall and disrupting its permeability [10]. Cell proliferation is inhibited by the interaction of nanoparticles with enzymes, proteins or DNA of microbes [[11], [12], [13], [14]].

Metallic nanoparticles have been found to useful in many applications such as biomedicine [15,16], catalysis [17], antimicrobial agent [18], antiplasmodial agent [19], textile engineering [20], drug delivery [21] etc. They can be synthesized by physical, chemical and biological methods. Physical and chemical methods are expensive and require toxic chemicals that can be adsorbed onto the surface of nanoparticles making them unsafe for biomedical applications. The biosynthesis of nanoparticles which includes synthesis by microbes [22], enzymes [23], algae [24] and plants [25] is advantageous over physical and chemical methods because it does not involve any toxic chemicals, easy to scale up and environment friendly. Unlike to microbial and enzymatic synthesis, plant extract mediated synthesis is simpler as it eliminates the process of maintaining cultures. The synthesis of silver nanoparticle from different parts of plants has been described by various researchers for many years. The synthesis of silver nanoparticles by reacting aqueous root extracts of *Rubus ellipticus* with 1 mM silver nitrate solution was reported by Khanal et al. [26]. The antifungal activity of silver nanoparticles synthesized from bamboo leaf extract was assayed against *Bipolaris maydis, Exserohilum turcicum,* and *Curvularia lunata* [27]. The Fenugreek leaf extract was found to contain certain bioactive molecules that led to the reduction of silver ion to their nano form. These nanoparticles showed potential antibacterial activity against *S. aureus, E. coli, P. aeruginosa* and *V. cholera* [28]. The biosynthesized nanoparticles have been shown to be a good antimicrobial agent. The stability of nanoparticles over a longer period of time must be assured.

*P.longifolia* is a sublime evergreen plant, native to India and is widely planted fofr its effectiveness in relieving noise pollution. The alkaloids isolated from stem bark extract were found to have good antimicrobial and cytotoxic activity [29]. In traditional medicines various herbal preparations being used to treat duodenal ulcers. The plant has been used in traditional medicine to treat fever, skin diseases, diabetics, anti-aging, hypertension and helminthiasis [30,31]. The bark of *P. longifolia* has also been found to have some therapeutic importance. It is used to lower blood pressure, stimulate breathing, and help with fever and skin diseases, diabetes, high blood pressure, and Vata and Pitta disorders. It contains secondary plant substances such as flavonoids, alkaloids, steroids and carbohydrates. The flavonoids isolated from *P. longifolia* bark extract showed promising results against various microorganisms such as *Bacillus subtilis, Bacillus thuringiensis, Escherichia coli, Pseudomonas aeruginosa* and *Candida albicans, Aspergillus niger* compared to standard drugs (penicillin, gentamicin and ketoconazole) [32]. Similar results were found when *P. longifolia* bark stem extract was tested against *Escherichia coli, Bacillus subtilis, Salmonella typhi, Proteus mirabilis, Pseudomonas aeruginosa, Klebsiella* and *Staphylococcus* *aureus* [33]. The methanolic extract of *P. longifolia* was used to synthesize copper-based nanoparticles and proved to be highly effective against *S. aureus, E. coli* and *C. albicans* fungi [34].

In the present investigation, we synthesized silver nanoparticles using *P. longifolia* leaf extract and confirmed them by characterization with different analysis techniques such as UV–visible, DLS, XRD, TEM, SEM and EDX. Subsequently, the synthesized nanoparticles were tested for their *in vitro* antifungal activity against *A. alternata*, a phytopathogenic fungus, by assessing their MIC and MFC and cytomorphological changes in fungal spores.

## 2 Material and methods

### 2.1 Chemicals

All chemicals used in the experiment are reagent grade and were purchased from Sigma-Aldrich Co. (St. Louis, MO, USA) and Hi-media Lab Pvt. Ltd. (Mumbai, India). A pure culture of phytopathogenic fungi *Alternaria alternata* (ITCC No. 6134), was obtained from Indian Institute of Agricultural Research, New Delhi, India.

### 2.2 Collection of plant and preparation leave extract

Leaves of *P. longifolia* were collected from Botanical Garden, University College of Science, Udaipur, Rajasthan, India and authenticated by Herbarium at Department of Botany, University of Rajasthan, Jaipur, Rajasthan, India (RUBL211518). The fresh leaves were thoroughly washed with tap water to remove dust particles, followed by deionized water, dried at room temperature and cut into small pieces. To prepare an extract, 20 g of finely chopped leaves were mixed in 125 ml of deionized water and boiled at 100 °C in a serological water bath for 5 min. The solution was filtered using Whatman filter paper 3 and then with a vacuum filtration unit using cellulose nitrate membrane filters (pore size of 0.2 μm). The prepared extract was stored at 4 °C for further use.

### 2.3 Synthesis of PL-AgNPs

**Fig. 1:**
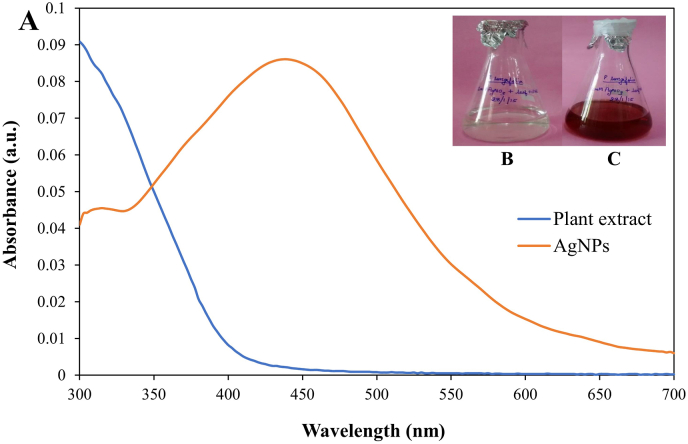
**A.** UV–visible spectrum of plant extract and AgNPs, **B**. before, **C**. after reduction.

The PL-AgNPs were formed by adding 3 ml of plant extract to 60 ml of 1 mM silver nitrate solution at room temperature. The reaction was performed in the dark to avoid photoactivation of silver nitrate. The reduction of AgNO_3_ to AgNPs was observed after 24 h and indicated by its characteristic reddish-brown colour (Fig. 1C).

### 2.4 Qualitative phytochemical screening of aqueous leaves extracts of *P. longifolia*

Qualitative testing of the phytochemicals for alkaloids, carbohydrates, glycosides, saponins, phenols and proteins were performed in the extracts using the standard method outlined in Table 1 [35].

**Table 1:** Qualitative phytochemical analysis of crude aqueous extract of P. longifolia.

S. No	Phytoconstituents	Name of tests	Indication	Aqueous extract
1	Alkaloids	Mayer's reagent	Reddish-brown ppt. or colouration	+
2	Carbohydrates	Molish's test	Violet ring at the junction	+
3	Tannins	Braymmer's test	Blue or greenish colour	+
4	Flavonoids	Alkaline reagent	colourless	+
5	Phytosterols	Salkowski's test	Red colour	+
6	Volatile oils	Sudan III	Red colour	+
7	Saponins	Foam test	Layer of foam at the surface	+

### 2.5 Optimization of synthesis of PL-AgNPs

To evaluate the optimal conditions for the synthesis of nanoparticles, various parameters such as concentration (1 mM, 2 mM, 3 mM, 4 mM and 5 mM), temperature (4 °C, 30 °C, 60 °C and 90 °C C), pH (3, 5, 7 and 9) and time (1 h, 2 h, 3 h and 24 h) are considered. Different temperatures were maintained by storing the nanoparticle solution in refrigerator at 4 °C and in ovens at 30 °C, 60 °C, 90 °C for 24 h. The pH of the solutions was adjusted with 0.1 N HCl and 0.1 N NaOH. The effect of these parameters on nanoparticle synthesis was analyzed using a UV–Vis spectrophotometer.

### 2.6 Characterization of PL-AgNPs

UV–Vis analysis was performed by diluting the sample and scanning between 300 and 700 nm using deionized water as a reference (Fig. 1). Necessary precautions have been taken to maintain sterility conditions in order to obtain effective, accurate and contamination-free results. The PL-AgNPs solution thus obtained was purified by repeated centrifugation at 15,000 rpm for 20 min, followed by re-dispersing the pellet of PL-AgNPs three times in deionized water. After freeze drying (lipolysed) the purified silver particles, the structure and composition were analyzed by DLS, XRD, SEM, TEM and EDX and further studied for their antimicrobial assay.

The particle size and nature of the PL-AgNPs were determined by XRD. This was performed using a Rigaku Ultima IV X-ray diffractometer with voltage and current range of 40 kV and 40 mA respectively. Cu kα with a wavelength of 0.154 nm was used. The material used for the analysis was finely ground, and the average bulk composition was determined. The scanning was done in the region of 20°–90° with step size of 0.02. The peaks obtained were corroborated with the Joint Committee on Powder Diffraction Standards (JCPDS) library to account for the crystalline structure. The particle or grain size of the PL-AgNPs was determined using the Debye Scherrer's equation

Crystalline Size D = Kλ / Bcos θ

Where D, crystalline size Ǻ; K, crystalline-shape factor (0.9); λ, X-ray wavelength (1.540598); θ, observed peak angle degree; B, X-ray diffraction broadening (Full Width at half maximum) radian.

The AgNPs samples were vortexed and then transferred to a 1.0 ml zeta potential cuvette using thermal system DT-60H thermal analyzer (TGA-DTA) from MNIT, Jaipur, Rajasthan. (DTS1060, Malvern). The zeta potential of AgNPs was evaluated using a Zetasizer Nano ZS (Malvern Instruments Ltd, Malvern, UK) and the zeta potential was calculated using Henry's equation.

Analysis with a transmission electron microscope (TEM) was performed using the TEM instrument Tecnai 20 from IIT SAIF, Bombay. Scanning electron microscopy (SEM) and energy dispersive X-ray spectroscopy (EDX) analysis was performed using the XL 30 FE-SEM scanning electron microscope with EDX. The powder sample was taken directly onto an aluminum stub containing a carbon tap on the top surface and was stored in the instrument for analysis.

### 2.7 Antifungal assay of PL-AgNPs

Antifungal assay of biosynthesized nanoparticles was tested against *A. alternata* by Poison food method [36]. A stock solution of 500 ppm was prepared by dissolving 0.025 g of nanoparticles in 50 ml deionized distilled water and was kept for sonication till the homogenous solution was prepared. From this stock solution 10 ml of 100 ppm, 200 ppm, 300 ppm and 400 ppm solutions were prepared. In the same way 100–500 ppm solutions of silver nitrate, Mancozeb and Bavistin were also prepared and were used as control. Aqueous leaf extracts of *P. longifolia* was also used for antimicrobial assay to study the comparative analysis of antifungal activity of the extract against synthesized PL-AgNPs. 1 ml of test solution was added to 9 ml sterilized potato dextrose agar and then was poured in Petri plate. Further 6 mm inoculum disc of 7 days old culture was place at the center of solidified agar, aseptically and kept in incubator at 25 ± 2 °C. Average growth diameter was calculated after 7 days. Percent inhibition rate of mycelia was calculated using the formula given by Vincent [37].

$$\text{Inhibition rate \% }=\frac{\text{mycelial growth in control}-\text{ mycelial growth in treatment }\;}{\text{mycelial growth in control}}\times 100$$

### 2.8 Minimum inhibitory concentration and minimum fungicidal concentration of synthesized PL-AgNPs were determined

MIC was determined by broth dilution method [38]. 1000 ppm stock solution of nanoparticle was prepared by mixing 10 mg PL-AgNPs in 10 ml sterilized water and was kept for sonication till the homogenized solution was prepared. Further two-fold serial dilution was done by mixing 5 ml of 1000 ppm solution with 5 ml sterilized potato dextrose broth (PDB) to form 500 ppm solution. In the same way 250 ppm, 125 ppm, 62.5 ppm, 31.25 ppm, 15.62 ppm and 7.81 ppm (PDB) solutions were prepared which were then inoculated with 100 μl spore suspension (1 × 10^6^ spores/ml) of *A. alternata.* Further the tubes were kept in shaking incubator at 25 ± 2 °C for 72 h. PDB with only inoculum was considered as control. Optical density of PDB solutions with inoculum was taken to observe the growth. This experiment was done in triplicate in separate repeats. To determine MFC a loop full of fungal biomass from all concentrations were streaked on the sterile slants of potato dextrose agar (PDA) and were incubated at 25 ± 2 °C for 72 h after which the growth was observed. Appearance of growth indicates that the extract concentration is fungistatic and absence of growth indicates that extract concentration is fungicidal.

### 2.9 Effect of PL-AgNPs on morphology of test fungi

Effect of nanoparticles on various morphological and cytological parameters of test pathogen was studied. Test fungi were treated with increasing concentrations of the nanoparticles up to MIC. A small fungal biomass consisting of mycelium and conidia was taken from each tube and microscopic examination was done after staining with cotton blue and mounting in lactophenol. Change in mycelium width, conidia size, and conidiophore morphology were observed with the help of Olympus trinocular research microscope BX-51 and analyzed using ocular microscopy.

## 3 Results and discussions

### 3.1 Synthesis of PL-AgNPs

After mixing plant extract with 1 mM silver nitrate solution the colour change was observed from yellow to dark brown in *P. longifolia* (Fig. 1C), which indicated the formation of nanoparticles. This physical appearance of the reaction mixture turning from yellow to reddish-brown is due to the surface plasmon resonance of the PL-AgNPs, which is considered to be the primary indication of nanoparticle formation. Similar results have been observed in *Enicostema axillare* [39], *Coleus aromaticus* [40] and *Ceratonia silique* [41].

### 3.2 Phytochemical screening

**Fig. 2:**
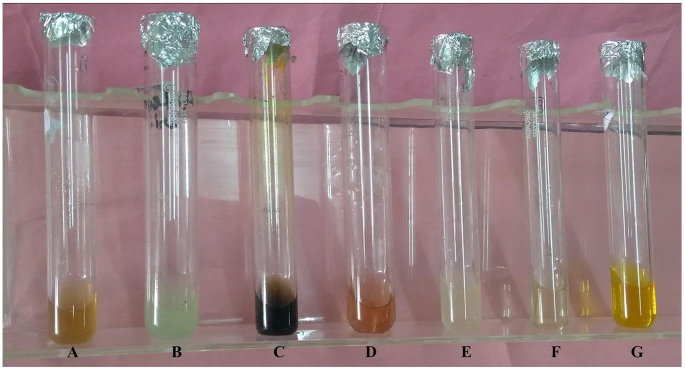
*P. longifolia* aqueous leaf extract phytochemical screening **i.** Alkaloids, **ii.** Carbohydrates, **iii.** Tannins, **iv.** Volatile Oils, **v.** Phytosterol, **vi.** Saponins and **vii.** Flavonoids.

For the synthesis of metallic nanoparticle phytochemicals are the most potent materials for biological activities. Soluble phytochemicals such as polyphenols, flavones, organic acids and quinones are greatly responsible for immediate reduction [42]. The phytochemical screening of *P. longifolia* aqueous extract revealed that it includes alkaloids, volatile oils, and saponins, which appear to be responsible for nanoparticle formation (Table 1, Fig. 2). The presence of carbohydrates, flavonoids, steroids, terpenoids, phenolics, tannins and glycosides were reported in the previous study [43].

### 3.3 Characterization of PL-AgNPs

#### 3.3.1 UV–visible spectrophotometer

After scanning the samples between 300 and 700 nm on UV-visible spectrophotometer, it was observed that rapid bio-reduction of silver nitrate was achieved using *P. longifolia* extract as reducing agent. The UV–visible spectrum was observed to be around 435 nm (Fig. 1). These biosynthesized nanoparticles were taken further for characterization.

#### 3.3.2 Optimization of PL-AgNPs

**Fig. 3:**
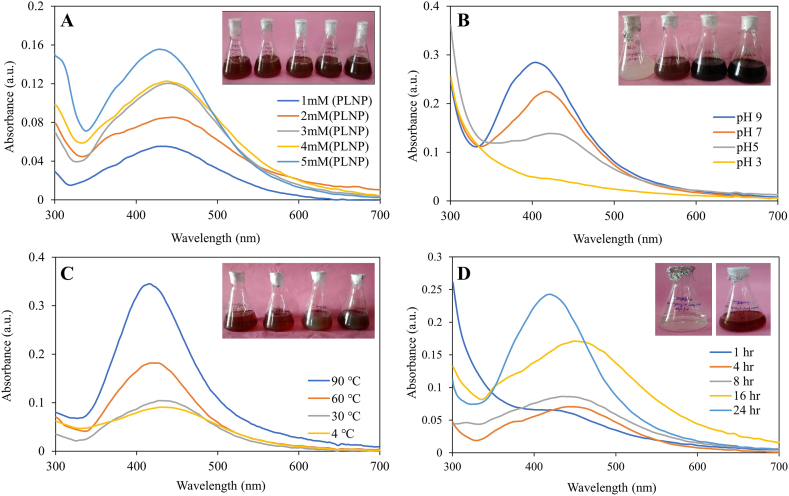
Optimization of synthesis of PL-AgNPs at different **A.** Concentration of AgNO_3_, **B.** pH, **C.** Temperature **D.** Time.

*Concentration:* It has been observed that at the concertation of 1 mM AgNO_3_ show rapid colour change from yellow to reddish brown with high stability. Whereas the increase in concentration of AgNO_3_ (2 mM, 3 mM, 4 mM and 5 mM) for nanoparticle synthesis resulted in agglomeration of nanoparticles and reduced the stability. However, resulting a shift in peak was recorded (Fig. 3A). Therefore, 1 mM concentration of AgNO_3_ was taken as optimum concentration having maximum wavelength and stability at 430 nm wavelength. These results were in agreement with the earlier investigations made by Rajendran et al. [44]. They found that a concentration of 1.0 mM silver ion was optimum for the production of AgNPs utilizing *Annona squamosa* peel extract.

pH*:* pH plays an important role in nanoparticle synthesis. At pH 3, no recognizable peak was found in the case of *P. longifolia* leaves mediated nanoparticle formation, however as pH increased, the wavelength increased and the absorbance dropped (Fig. 3b). At 9 pH a sharp peak was observed at 403 nm wavelength but the nanoparticles formed were not stable and agglomeration was observed at the base of the flask. At 7 pH the nanoparticles formed were found to be highly stable and a sharp absorbance peak was observed at 418 nm, thus 7 pH was taken as optimum for nanoparticle synthesis through *P. longifolia* leaves extract. Sun et al. [45] observed that the synthesis and aggregation of AgNPs was sluggish at acidic pH whereas, Edison & Sethuraman, [46] reported that Ag^+^ might precipitate as AgOH at basic pH. They also found that the optimum condition for the preparation of AgNPs using *Terminalia chebula* was at pH 7.0. Similar results were also observed by Krishnaraj et al. [47], a pH of 7.0 was ideal for the synthesis of AgNPs using *Acalypha indica* leaf extract.

*Temperature:* The synthesis of PL-AgNPs at pH 7 was further optimized at various temperature such 4 °C, 30 °C, 60 °C and 90 °C. Figure 3C shows enhance the nanoparticle synthesis as the temperature increased. Although, at the high temperature (90 °C) a sharp band was observed, nanoparticles were highly unstable, agglomerated and get settle down at the bottom. While at the low temperature (4 °C) no optimum peak was observed. The formation of the nanoparticles was observed between 30 and 60 °C. However noticeable colour change was observed from pale yellow to dark brown for AgNPs at 430 nm at 60 °C. Further, at 60 °C the peaks were sharp (Fig. 3C). In the previous study, Birla et al. [48] reported that among the various temperature optimized for nanoparticles synthesis using plant extract, 40–60 °C temperature supported the formation of nanoparticles with high stability.

*Time:* The magnitude of the yield of nanoparticles depends on the length of the reaction time, *i.e*., the duration of the interaction of the silver salt with the plant extract [49]. Increasing the time of reaction resulted in increased colour intensity with the duration of incubation and gradual increase in the absorbance spectrum with surface plasmon resonance at 432 nm (Fig. 3D). The maximum absorbance was observed at 24 h. Even after 2 months of incubation in a dark chamber at room temperature, the stability of PL-AgNPs biosynthesized in this study revealed that the there was no alteration in the peak.

Thus, in this report the nanoparticles synthesized using 1 mM silver nitrate solution and *P. longifolia* leaves extract when kept at temperature 60 °C, pH 7 and after 24 h of incubation showed a sharp peak at 413 nm.

#### 3.3.3 DLS analysis of PL-AgNPs

**Fig. 4:**
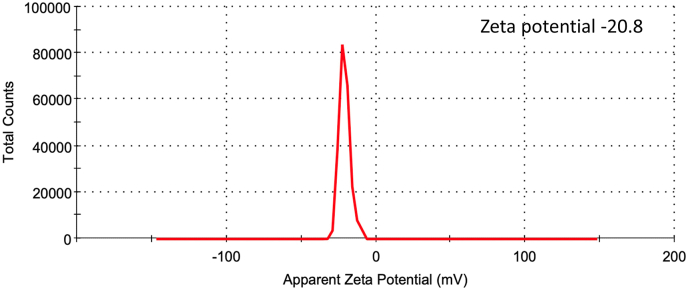
Zeta potential of synthesized PL-AgNPs.

Using the DLS technique, the surface zeta potential of synthesized AgNPs in aqueous colloidal solution was measured. The negative zeta potential was determined to be −20.8 mV in this investigation, with a zeta deviation of 5.42 mV (Fig. 4). The zeta potential range of ±30 mV is thought to be the most stable for AgNPs [50]. The high negative value indicated that synthesized AgNP did not agglomeration and remained suspended. In our case AgNPs solution remain suspended and stable for more than 2 months [51].

#### 3.3.4 XRD analysis of PL-AgNPs

**Fig. 5:**
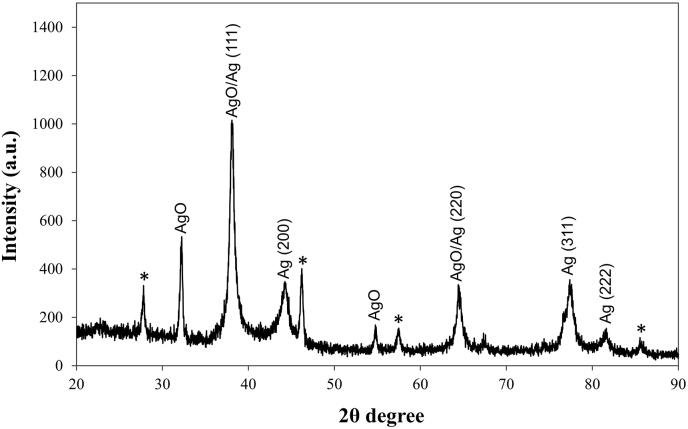
X-ray diffraction of synthesized PL-AgNPs.

Fig. 5 shows X-ray diffraction pattern of *P. longifolia* mediated nanoparticles which suggested that the sample is not in single phase. The presence of Ag and AgO were identified through Joint Committee on Powder Diffraction Standards (JCPDS) card no 89–3722 and 76–1489 respectively. The crystallite size of nanoparticles was also calculated by Scherer's Formula (Table 2). The obtained values of nanoparticles were found to be between 17 and 48 nm. The maximum intense peak is found at 38° due to Ag phase which suggest that it possess large amount of nano Ag particles. Generally, the broadening of peaks in the XRD patterns of solids is attributed to particle size effects. Broader peaks signify smaller particle size and reflect the effects due to experimental conditions on the nucleation and growth of the crystal nuclei [52]. The peaks at five different facets of silver namely (111), (200), (220), (311) and (222) planes correspondence to their 2q, such as 32.25°, 42.20°, 54.76°, 77.37^o^ and 81.60^o^, consistent with the faced center cubic (FCC) crystal structure of PL-AgNPs, the similar observation was also reported in the study by Tripathy et al. [53]. A few unassigned peaks were also noticed in the vicinity of the characteristic peaks might be due to the crystallization of the biomolecule on the surface of the AgNPs [54]. Few unassigned peaks might have resulted due to the capping agent stabilizing the nanoparticle [49]. Independent crystallization of the capping agents was ruled out due to the process of centrifugation and redispersion of the pellet in deionized water after nanoparticle formation as part of purification process. Therefore, XRD shows mixed phase behavior.

**Table 2:** XRD analysis of PL-AgNPs through Scherer's formula.

Phase name	hkl	Intensity	2θ	FWHM (2θ)	B cos θ	Crystal Size (nm)
AgO	111	51	32.25	0.3843	0.003216	42 nm
AgO/Ag	200/111	100	38.07	0.7510	0.006157	22 nm
Ag	200	34	42.20	1.0147	0.008142	17 nm
AgO	220	17	54.76	0.3746	0.002910	48 nm
AgO/Ag	311/220	33	46.40	0.7805	0.005788	24 nm
Ag	311	35	77.37	1.2102	0.008236	17 nm
Ag	222	14	81.60	1.0421	0.006908	20 nm

#### 3.3.5 SEM, EDX and TEM analysis of PL-AgNPs

**Fig. 6:**
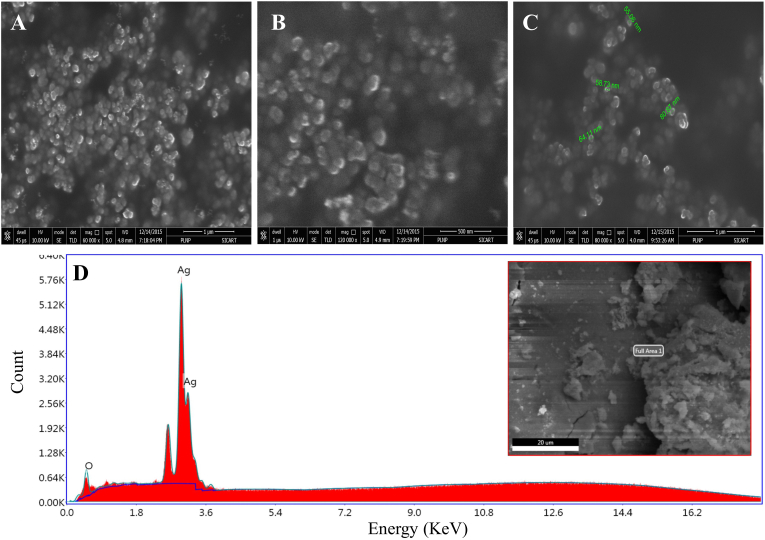
**A-C.** SEM micrograph of synthesized PL-AgNPs, **D.** EDX spectrum of elemental composition of PL-AgNPs.

**Fig. 7:**
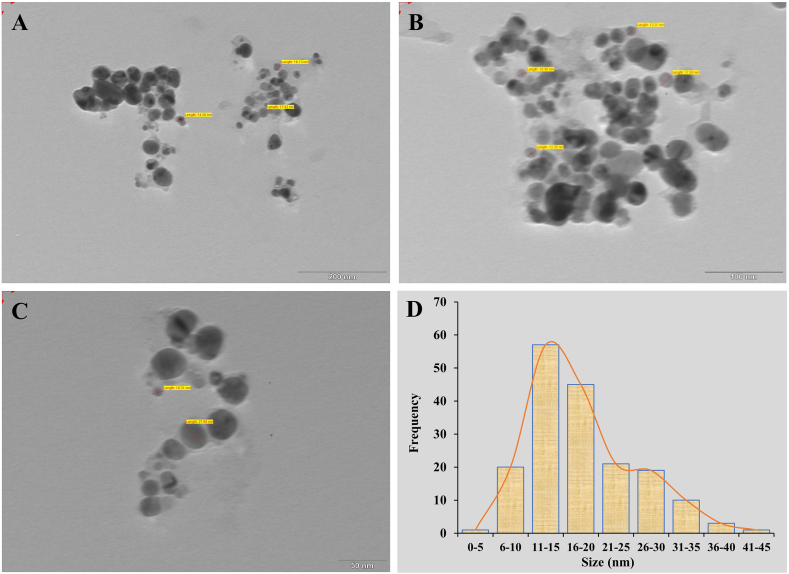
**A-C.** TEM micrograph of synthesized PL-AgNPs, **D.** Histogram of particles size.

After analyzing the SEM images (Fig. 6) of PL-AgNPs synthesized from leaf extract of *P. longifolia* it can be said that they are spherical in shape and their size less than 18 nm. EDX analysis of PL-AgNPs synthesized from *P. longifolia* shows that it contains 72% silver and 28% oxygen and remaining percentage was different metal atoms such as Na, P, S and Al in trace amounts (Fig. 6D). EDX analysis confirmed the presence of silver, while the oxygen signal indicated that the extracellular organic material was probably adsorbed on the PL-AgNPs surface [55]. The mean size of PL-AgNPs according to histogram image analysis of Transmission electron microscopy images is approximately 14 nm (Fig. 7D). The shape and size of nanoparticles varies from plant to plant and part used and also the phytoconstituents like alkaloids, flavanoids, tannins and cardiac glycoside present in them at the time of synthesis [56]. TEM results also revealed that particles were in spherical shaped and size of the particles were obtained between range of 10–30 nm (Fig. 7A–C). In situ TEM analysis with high spatial resolution has been used to observe and analyze defects within piezoelectric nanocrystals [57]. This technique is also very efficient and powerful medium for energy conversion, transportation, food production and environmental protection in gaseous environment [58].

#### 3.3.6 Antifungal activity of PL-AgNPs against *A. alternata*

**Fig. 8:**
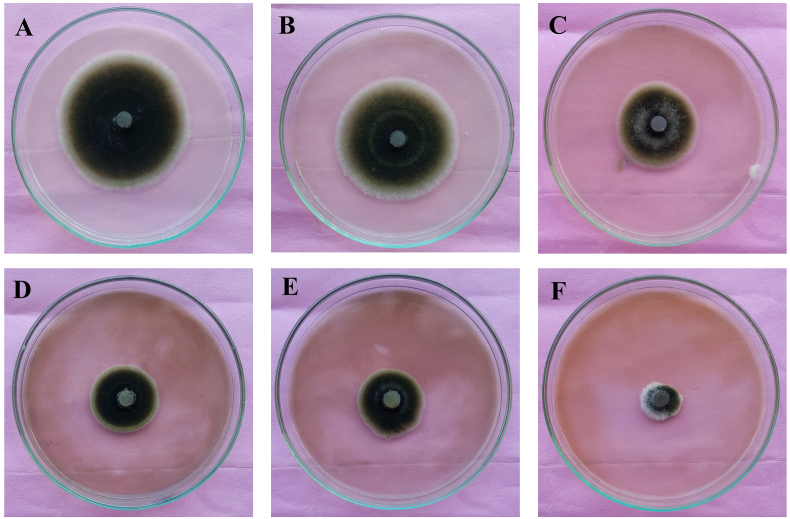
Antifungal activity of PL-AgNPs against *A. alternata***A.***P. longifolia* aqueous leaves extract, **B.** 100 ppm PL-AgNPs, **C**. 200 ppm PL-AgNPs, **D.** 300 ppm PL-AgNPs, **E.** 400 ppm PL-AgNPs, **F.** 500 ppm PL-AgNPs.

Mycelial radial growth against *A. alternata* was used to assess the antifungal activity of the produced AgNPs (Table 3). Nanoparticle solutions at various concentration (500 ppm, 400 ppm, 300 ppm, 200 ppm and 100 ppm) assayed *in vitro* against fungal culture (Fig. 8). Plant extract was also used for antifungal treatment as control with nanoparticles. It was observed that PL-AgNPs at 500 ppm showed maximum inhibition of mycelial growth at 81%. mycelial growth inhibition was observed at 500 ppm PL-AgNPs (81%). It showed a significant inhibition in comparison to aqueous leaf extract (53.4%) and silver nitrate solutions. Thus, it was observed that *P. longifolia* mediated PL-AgNPs showed significant antifungal activity against *A. alternata.* Similar findings were observed in the study by Kumar et al. [59], the antimicrobial assay of plant extract, silver nitrate, fungicide and biosynthesized nanoparticles were assayed against *A. solani.* Abdelmalek et al. [60] also reported the potent antifungal activity of PL-AgNPs against *A. alternata.* Changes in the structure of fungal cells could be one of the regions for AgNPs action. Furthermore, nanoparticles have the potential to disrupt macromolecule (DNA and protein), resulting in fungal death [[61], [62], [63]].

**Table 3:** Mycelial growth inhibition activity of PL-AgNPs against Alternaria alternata

S. No.	Treatment	Mycelial growth inhibition (%)
1	Control with water	00.00 ± 00
2	Plant extract (control)	27.33 ± 0.54
3	AgNPs 100 ppm	36.78 ± 1.15
4	AgNPs 200 ppm	59.77 ± 1.15
5	AgNPs 300 ppm	64.36 ± 0.57
6	AgNPs 400 ppm	71.26 ± 0.57
7	AgNPs 500 ppm	81.03 ± 1.00

#### 3.3.7 MIC and MFC of PL-AgNPs

**Fig. 9:**
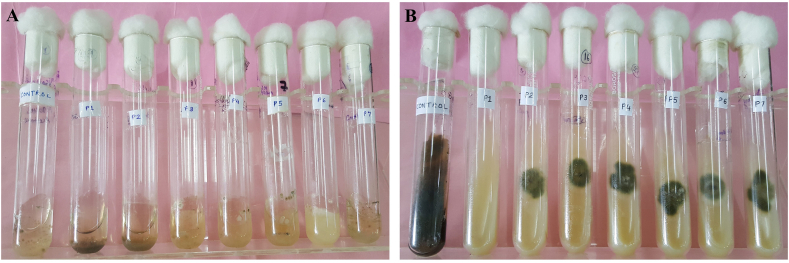
MIC and MFC of PL-AgNPs (**A).** Minimum inhibitory concentration, Control, **P1**: 500 ppm, **P2**: 250 ppm, **P3**: 125 ppm, **P4**: 62.5 ppm, **P5**: 31.25 ppm, **P6**: 15.62 ppm, **P7**: 7.81 ppm. (**B).** Minimum fungicidal concentration, Control, **P1**: 500 ppm, **P2**: 250 ppm, **P3**:125 ppm, **P4**: 62.5 ppm, **P5**: 31.25 ppm, **P6**: 15.62 ppm, **P7**: 7.81 ppm.

The MIC was defined as the lowest concentration of silver nanoparticles that caused decrease in absorbance compared with that of the control. Results were compared by taking absorbance at 595 nm using ELICO UV–vis spectrophotometer [64]. The MIC and MFC of nanoparticles synthesized from leaves extract of *P. longifolia* were observed against *Alternaria alternata* are illustrated in Fig. 9A&B. After analyzing the spectrophotometric absorbance of PL-AgNPs solutions it was observed that at 500 ppm, 250 ppm the absorbance was −0.042 and −0.032 respectively whereas at 125 ppm, 62.4 ppm, 31.25 ppm, 15.62 ppm and 7.81 ppm the absorbance was found to be 0.176, 0.237, 0.413, 0.626 and 0.825 respectively. The absorbance of control having PDB with only inoculum was found to be 1.413. Thus, MIC and MFC of PL-AgNPs was found at 125 ppm and 500 ppm respectively. The determination of MIC is necessary for prescribing its appropriate dose, as unnecessarily high dose of nanoparticles may cause considerable harm to the quality of the commodity treated [65]. In the present study, the nanoparticles exhibited fungistatic nature at MIC against the test fungi, but at higher concentrations it became fungicidal. The spectrophotometric study proved that with the decrease in concentration of nanoparticle solutions the fungal biomass is increased which has resulted in increase in its absorbance. Bahrami-Teimoori et al. (2017) observed the same type of results against *A. alternata* [57].

The MIC was defined as the lowest concentration of silver nanoparticles that caused 90% decrease in absorbance compared with that of the control (without test compound). MIC_90_ was determined in triplicate by broth dilution method [21], and the average value was considered. After 48 h of incubation, 5 and 10 μl from each visually clear test tube were plated onto freshly prepared agar YEPD plates. These plates were further incubated to check the MFC. Results were compared by taking OD at 595 nm using Mecasys Optizen 3220-UV spectrophotometer.

#### 3.3.8 Cytomorphology

Effect of PL-AgNPs on mycelial width, conidia length and width of *A. alternata* are presented in Table 4. A gradual decrease in conidia size and increase in hypha width with increase in concentration of nanoparticle was observed in both nanoparticle solutions. Mycelial width of *A. alternata* increased up to 69.1% at 125 ppm concentration PL-AgNPs. Conidia size of the *A. alternata* was reduced up to 75.7% at 125 ppm concentration of PL-AgNPs. With the increase in concentration the number of conidia formed were reduced and at higher concentrations no conidia formation was observed. The increase in hyphae with could be due to the uptake of NPs by the fungal hyphae [66]. Similar to this, Cu6-PLGA NP absorption has been seen in *Aspergillus flavus* fungal cells in the past. In instance, Patel et al. [67] demonstrated that the size of the NPs has a significant impact on the absorption of Cu6-PLGA NPs into *A. flavus* spores and mycelium. In these studies, NPs connected with the surfaces of fungal cells and, after 1 h of incubation, were effectively internalised. In this study, we demonstrated that PL-AgNPs interacted with and penetrating into the conidia and hyphae of *A. alternata*, increasing the absorption of the encapsulated substance into fungal cells, with the objective of preventing spore germination and subsequently fungal infection.

**Table 4:** Effect of PL-AgNPs on mycelial width and conidial size of Alternaria alternata.

PL-AgNPs concentration	Mycelial width	% Increased in mycelial width	Conidia size	% Decreased in conidia size
Control	9.167 + 0.29	–	876.35 + 1.67	–
7.81 ppm	10.33 + 0.28	11.29	862.56 + 2.13	1.57
15.62 ppm	14.5 + 0.5	36.78	636.87 + 2.25	27.32
31.25 ppm	19.33 + 0.76	52.58	525.8 + 1.42	40
62.50 ppm	25.5 + 0.5	64.05	347 + 1.77	60.4
125 ppm	29.6 + 0.28	69.10	212.94 + 1.31	75.7
250 ppm	NF			
500 ppm	NF			

## 4 Conclusion

In the present research investigation, leaf extract of *P. longifolia* was used to synthesize PL-AgNPs and it was efficiently able to reduce silver nitrate to their nanoparticles. The synthesis method is benign, inexpensive and environmentally friendly. The synthesized nanoparticles were tested for their effects on *A. alternata* and found to be highly active against the fungus. Thus, from the above study, it can be confirmed that PL-AgNPs synthesized from leaf extract of *P. longifolia* can act as a potent antifungal against *A. alternata* that causes leaf spot, rot and rot on many parts of plants, resulting in a reduction in their productivity. In addition, they can also be studied for their applications in enhancing the growth of crops.

## Authors contribution

A.D. and K.R. Execute the Experiment; Formal Analysis; Data Curation. S.R., Conceptualization; Methodology; Data Curation; Software; Validation; Figure Preparation. A.D., K.R. and S.R. Writing– Original Draft Preparation. K.S. Supervision. Finally, all the authors contributed in discussing, reviewing and approved the final version of manuscript for publication.

## Declaration of competing interest

The authors declare that they have no known competing financial interests or personal relationships that could have appeared to influence the work reported.
